# Addressing overcrowding in an emergency department: an approach for identifying and treating influential factors and a real-life application

**DOI:** 10.1186/s13584-020-00390-5

**Published:** 2020-09-02

**Authors:** Guy Wachtel, Amir Elalouf

**Affiliations:** Department of Management, Bar-Ilan University, 5290002 Ramat Gan, Israel

**Keywords:** Emergency departments, Overcrowding, Length of stay, New factors, Algorithm

## Abstract

**Background:**

Overcrowding in hospital emergency departments that arises from long length-of-stay is an unfortunate common occurrence. While some factors affecting length-of-stay are well known, there may be additional factors that have not yet been properly addressed. This research offers a method for emergency department managers to use available data from their departments to identify new factors that significantly influence emergency departments crowding and patient length-of-stay.

**Methods:**

We propose an algorithm that can assist emergency department managers in determining which of these factors to address, given budgetary constraints. We implemented it in a case study which takes into account factors that are known to be influential, e.g., reason for arrival, occupancy in the emergency department, and arrival time, as well as factors that are explored for the first time in this paper, such as patient heart rate, the number of accompanying escorts, and the number of tests assigned to patients (e.g., blood tests and urinalysis).

**Results:**

All the implemented and new factors are shown to have a significant influence on the length-of-stay and crowding. We also obtained additional support for our results by interviewing emergency departments physicians and nurses from various hospitals.

**Conclusions:**

It is expected that, by taking all the above factors into consideration, emergency departments efficiency can be improved. The algorithm constructed here allows the choice of the most cost-effective factors to be improved, subject to a given budget. We have been able to derive practical recommendations that emergency departments managers might use to limit crowding and patient length-of-stay.

## Introduction

A hospital’s emergency department (ED) is responsible for evaluating patients when they first arrive and subsequently assigning them to appropriate departments in the hospital or referring them to general practitioners or to specialists for further treatment. Long length-of-stay (LOS) and overcrowding in EDs are serious yet commonplace problems in health-care systems around the world, and in Israel in particular. In fact, major media outlets publish articles almost weekly on overcrowding in EDs, reporting ED patient occupancy of more than 200% [29]. To handle overcrowding, many hospitals determine a maximum number of patients who can occupy the ED and, once this level is exceeded, refer incoming patients and ambulances to other EDs in the area, and even outright reject non-urgent cases. Clearly, these solutions are highly inconvenient for patients and result in a loss of income for hospitals and EDs.

Appropriate resource management, with a scheduling-and-control approach, could speed up patient handling procedures. This would reduce the amount of time that patients spend in the ED, thereby improving service quality, increasing patient throughput. To develop an effective resource management approach, it is first necessary to identify the dominant factors that influence a patient’s length-of-stay and crowding in an ED. Many of these factors are well known and have been empirically studied, including, for example, non-urgent visits, so-called “frequent-flyer” patients, low staffing and resource levels, and time of year (e.g., crowding is expected during the influenza season) [22]. Other factors, however, have yet to be explored. One potentially influential factor is the presence of escorts who accompany patients. On the one hand, an escort might help to streamline the processing of the patient he or she is accompanying, by helping to explain the patient’s condition or by walking with the patient to various tests. On the other hand, the mere presence of escorts in the ED may interfere with the capacity of physicians and other staff members to work and move in the space. In addition, a patient’s heart rate upon arrival might provide information on his or her projected length-of-stay. A patient’s heart rate constitutes a quantifiable indication of his or her physical and mental state, which may reflect whether the patient requires further surveillance in the ED. For that reason, heart rate is also part of the emergency severity index (ESI) calculation method and is an early warning sign [34]. Once factors influencing ED crowding and patient length-of-stay are identified, scheduling-and-control models can be developed in order to predict changes in the availability of resources in real time and to indicate which steps should be taken to ensure that the ED provides more efficient service.

The goal of this research is to show that, by collecting data inside an ED, we can identify new factors that have the potential to influence ED crowding and patient length-of-stay. Then, given an initial set of candidate factors, we use statistical analysis to identify the factors whose effects are indeed significant. We further propose an algorithm that can assist ED managers in determining which of these factors to address, given budgetary constraints. The reader should keep in mind that we don’t use the observed factors to analyze or to predict the patients’ condition, but rather to give insights regarding the LOS and crowding situation that they cause. By that we aim to assist the ED’s management in allocating resources more effectively for the ED’s performances improvement. In a case study, we present an implementation of this procedure and derive practical recommendations that ED managers might use to limit crowding and patient length-of-stay.

### Literature review

Long length-of-stay in EDs has a negative influence on the quality of medical care that ED patients receive, as well as on hospital profits [23]. ED overcrowding and patient length-of-stay have therefore been attractive subjects for operations and health care researchers for many years, and numerous approaches have been developed to improve ED work flow. Most studies on ED work flow have focused on forecasting patient volume, scheduling physicians’ shifts and medical process chains, and planning resource utilization. These works have largely relied on three approaches: queuing and scheduling, mathematical and dynamic programming, and economics.

Numerous studies have used scheduling methods and computer simulations to identify means of increasing the efficiency of health care and reducing the amount of time that patients spend in the process chain. Syi, et al., [32], for example, used computer simulations to model an emergency medical services system, focusing on pre-hospital care. Broos, et al., [25] used an integrated nurse staffing and scheduling approach to analyze longer-term nursing staff allocation problems. They proved that staffing multiple nursing departments simultaneously, and integrating nurse characteristics into staffing decisions, can lead to substantial improvements in schedule quality in terms of cost, personnel job satisfaction, and effectiveness in providing high-quality care. Yariv, [26] built a simulation model, based on empirical data from five EDs in Israel, with the aim of identifying means of reducing turnaround time and improving ED service quality. The authors showed that a “fast-track” processing method, whereby “easier” patients are treated separately from others, was better than the common method in use at the time. They further showed that the model could be used to predict patient flow, length-of-stay, and crowding rate in the ED. Today, many hospitals in Israel use the fast-track model.

He, et al., [19] compared several scheduling strategies that are often discussed in the literature, including the fast track approach, first-in-first-out (FIFO) with priority, and physician and/or team triage. The authors concluded that adoption of triage physician and triage team strategies can enhance the overall performance of the ED. We intend here to propose a data analysis method that can assist the triage physician/nurse as a decision maker and improve ED performance even further. Ashour, et al., [2] recently proposed another scheduling approach, in which a dynamic grouping and prioritization (DGP) algorithm is used to identify the most appropriate criteria for classifying patients and prioritizing the corresponding groups according to their benefits to the system. Using a discrete-event simulation, the authors obtained statistical evidence that the DGP outperforms alternative strategies with regard to all criteria evaluated. Notably, however, implementation of this algorithm did not lead to any improvement in patient throughput.

One of the most frequently-cited articles on simulation models for ED operations is that of [13], which discusses the use of discrete-event simulation methods for advanced system-level investigation of ED operations. Similarly to [13, 26] used simulation models to evaluate the fast-track approach, showing that it decreased patients’ length-of-stay in the ED by tens of percentage points. Christine, et al., [14] also discussed discrete-event simulation approaches. Other papers analyzing patient length-of-stay are those of [18, 30]. These authors formulated multistage models describing the length-of-stay distribution for diverse patient groups, distinguished according to various factors such as diagnosis, severity of illness, age, or hospital. The researchers noted that information systems fail to capture certain types of information, and that there is a need to supplement the missing data. In this paper we attempt to do so by carrying out observations inside the ED. Brailsford, et al., [6] used empirical data from an ED to formulate a dynamics-based simulation model. They were able to predict future scenarios on the basis of simulated detection of bottlenecks. Brenner, et al., [7] carried out a similar simulation study at the Kentucky Chandler Hospital, with the goal of finding bottlenecks and helping management decide how to overcome them. Miro, et al., [27] studied the relations among processes involved in patient care and proposed a method for using empirical data and statistical analysis approaches to improve patient care and to enable the ED staff to perform their duties more efficiently and safely. A reorganization based on their findings was implemented, and successfully decreased patients’ length-of-stay in the ED.

Over the last few years, several studies have examined ED work flow using optimization and stochastic control techniques. Arun, et al., [8] proposed a stochastic control approach to prevent ED overcrowding. They used Petri-nets (PNs) to model patient and resource flow in the hospital system and proposed an optimal control policy that dictates when and how resources should be added or removed.

Weng, et al., [33] used simulations to optimize resource allocation in an ED. Their model incorporated an indicator called *NEDOCS* (National ED Overcrowding Study), which is calculated as follows (Eq. 1):

1 $$ NEDOCS=\left(\frac{P_{bed}}{B_t}\right)\times 85.8+\left(\frac{P_{admit}}{B_h}\right)\times 600+{W}_{Time}\times 5.64+{A}_{Time}\times 0.93+{R}_h\times 13.4 $$

This indicator is commonly used in the American medical industry to measure congestion in EDs (The authors also mentioned an alternative indicator of crowdedness, called *EDWIN*, which they did not use in their study). *NEDOCS* incorporates the following variables: *P*_*bed*_: the total number of patients in the ED at time *t*; *B*_*t*_: the total number of sickbeds in the ED at time *t*; *P*_*admit*_: the total number of patients waiting in the hospital at time *t*; *W*_*time*_: the amount of time, on average, during which a patient in the emergency room waits for a sickbed to become available; *A*_*time*_: the maximum amount of time that an emergency patient waits to be hospitalized; and *R*_*h*_: the number of emergency patients who use inhalation apparatus. The greater the value of the *NEDOCS*, the greater the degree of congestion (*NEDOCS* ≥ 100 means that there is overcrowding in the ED). Weng, et al., [33] showed that allocation of human resources has a substantial influence on the *NEDOCS* value. Anneveld, et al., [1] studied the accuracy of the *NEDOCS* tool by comparing it with the subjective feelings of the ED nurses and emergency physicians in an inner-city hospital in the Netherlands. They concluded that it is a reasonably good tool to quantify the impressions of overcrowding as experienced by the above medical staff in the ED. Herein, we adopt a modelling approach similar to that used by [33] and by [1]. In contrast to our model, however, their approach did not attempt to use specific variables to predict patient length-of-stay or crowding; the authors just measured crowding in specific time periods.

Another current in the literature uses algorithmic methods to derive recommendations for improving the quality of care in EDs. Most of these papers deal with the problem of increasing the accuracy of triage examinations. Berman, et al., [5] showed that computerized algorithms can serve as an effective tool for correcting patients’ characterizations in the triage, thus improving the examination process and the treatment that these patients receive. Chonde, et al., [9] and Fields, et al., [15] presented a prediction method for the estimation of patients’ Emergency Severity Index (ESI) levels and urgency. Ballard, et al., [4] had previously validated a similar algorithm for categorizing patients’ severity at the New York University ED. However, a recent study by [21] of the use of the ESI in Brazil proves that this tool must be used at least carefully: their results shew that despite rigorous and ongoing training of ESI users in the ED, a large number of patients were under - or over - triaged, see also [20, 28]. Advanced age, vital sign derangements (such as heart rate), and specific chief complaints were particularly under-appreciated when using the ESI algorithm. Ashour, et al., [2, 3] and Claudio, et al., [11] carried out additional studies in this field. These authors addressed the issue of triage algorithms that use Fuzzy Analytic Hierarchy Process (FAHP) and Multi-Attribute Utility Theory (MAUT) for ranking patients according to their characteristics. Using discrete-event simulations, they showed that, when addressing each ESI level separately, the new algorithm balances the length-of-stay and the time-to-bed waiting time. Claudio, et al., [10] also studied the latter method as a decision support model to be implemented together with new technology. They showed that the combination of this method and the technology enhances the capacity of triage nurses to prioritize patients waiting in the ED.

## Method

**Fig. 1:**
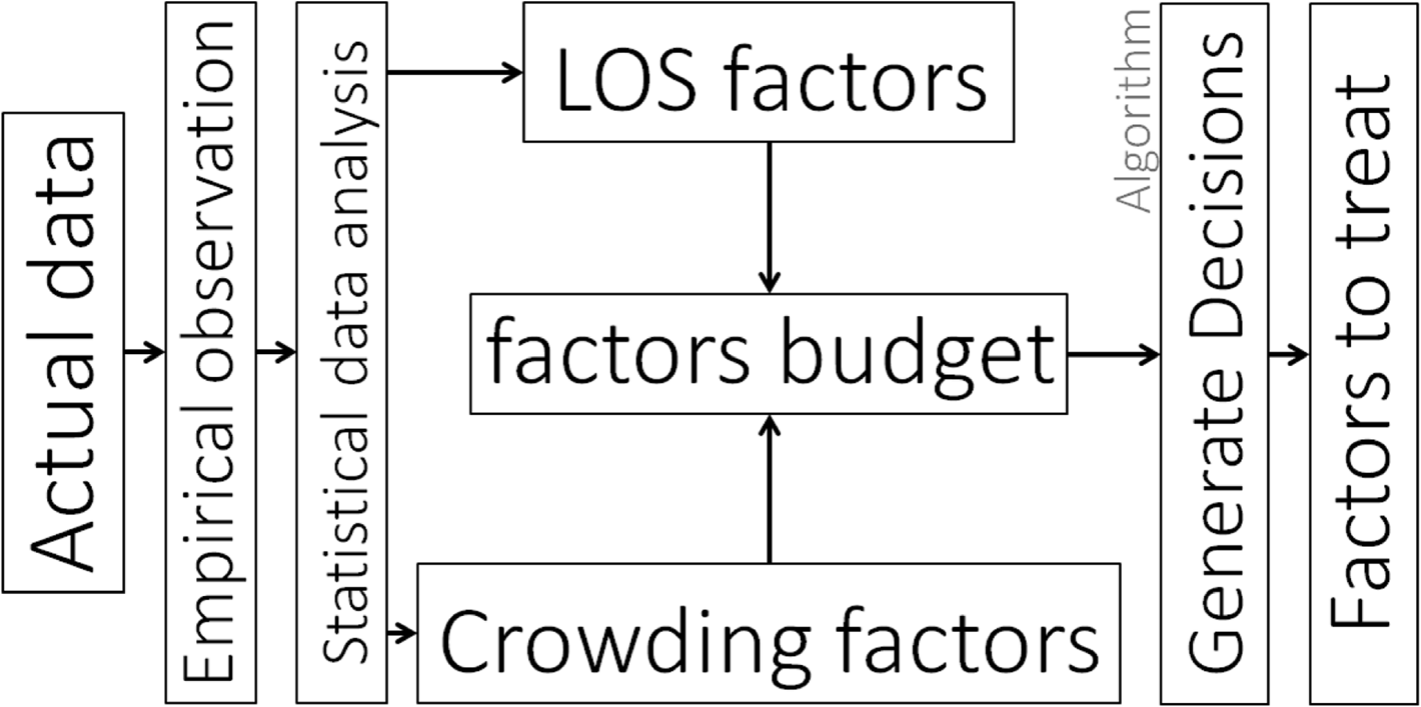
Decision making process-chain

As noted above, the goal of this research was to present a method for identifying previously unexplored factors that influence crowding and patient length-of-stay in EDs, and to suggest new work principles and methods that can guide ED managers. The method we propose is illustrated in the process flowchart shown in Fig. 1. Specifically, we first obtain empirical data from a real-life ED. These data can be collected from field observations and from information that is available to the management of the ED (e.g., exported data tables from the ED management software and from patients’ physical records). The different data sources are then synchronized. On the basis of a statistical correlation analysis on the collected data, we identify a set of treatable factors that might influence the variable of interest (i.e., patients’ length-of-stay or crowding). Statistical analysis is then carried out in order to identify the factors whose effects are indeed significant. Under the assumption that the ED management has a limited budget at its disposal for addressing each factor (a “factor budget”), each factor is then assigned a cost (which may be subjective and relative), based on the time, effort and financial cost associated with addressing it. As the identified number of factors may be very large, we then implement an algorithm (Algorithm 1), described in detail below, to rapidly identify which factors to treat. Specifically, the algorithm selects a subset of factors for further treatment, given the constraints of the “factor budget”, and taking into account each factor’s potential to reduce patient length-of-stay or crowding.

### The algorithmic solution

We address a problem with *n* factors that have been found to significantly affect patient length-of-stay, with the goal of identifying which factors among them the ED manager should address in order to reduce length-of-stay (and therefore crowding). Each factor *F*_*i*_ has two attributes: The first is *F*_*p*(*i*)_, 0 ≤ *F*_*p*(*i*)_ ≤ 1, reflecting the reduction in the current average patients’ LOS that can be achieved when *F*_*i*_ is addressed. Specifically, *F*_*p*(*i*)_ is the fraction by which *T* ≪ *LOS* is reduced, where *T* is the maximum fraction of time by which LOS can possibly be reduced. The second factor, *F*_*c*(*i*)_, is the cost of addressing *F*_*i*_. This cost can incorporate, for example, wages paid to ED personnel, costs of additional services (e.g., security at the entrance of the ED to limit the number of escorts and visitors), and costs of medicines.

The objective is to maximize the reduction of LOS, under a budget constraint (*B*). In order to find the optimal subset of factors that should be addressed, we represent any given subset of factors as π = (*F*_1_, *F*_2_, …, *F*_*k*_), where *F*_1_, *F*_2_ are the first and the second factors in π and not necessarily equal to *F*_(1)_, *F*_(2)_. Each set can be represented as a pair of measures, (*P*, *C*) where $P={\sum}_{F_i\in \uppi}\left(\left(1-{F}_{p(i)}\right)T\right)$ is the percentage of the potential reduction (*T*) that we did not utilize, and $C={\sum}_{F_i\in \uppi}\left({F}_{c(i)}\right)$ is the total cost.

We now outline a dynamic programming (DP) solution that entails constructing sets *S*_(*k*)_ of pairs (*P*, *C*). The sets in each *S*_(*k*)_ are arranged in increasing order of *P*-values. In order to restore the factor-set corresponding to a pair (*P*, *C*), each pair is assigned a unique code and a predecessor pair: ${\left(P,C\right)}_b^a$ where *a* is a unique code for the current pair and *b* is the code for the previous pair. Standard backtracking can then be used to identify the corresponding factor set.

If there are two pairs in *S*_(*k*)_, (*P*1, *C*1) and (*P*2, *C*2) such that *P*1 ≤ *P*2 and *C*1 ≤ *C*2, then the pair (*P*2, *C*2) is called “dominated” and may be discarded. The detailed pseudo-polynomial time DP algorithm is presented in Algorithm 1.

### Proposition 1

The complexity of the DP algorithm (Algorithm 1.) is *O*(*nB*).

### Proof 1

Since all *F*_*c*(*i*)_ and therefore all *C* are integers and the dominated pairs are discarded, there are at most *B* pairs in set *G*, *S*_(*k*)_. Furthermore, construction of *G* in lines 4–9 requires *O*(*B*) elementary operations, since *G* is constructed from a single *S*_(*k*)_. Discarding all the dominated pairs, line 9, is done in linear time (in the number of pairs, which is at most *B*). In Step 2, lines 3–11 are performed *n* times. Thus, the total complexity of the algorithm is *O*(*nB*).

#### Algorithm 1:The exact pseudo-polynomial DP algorithm


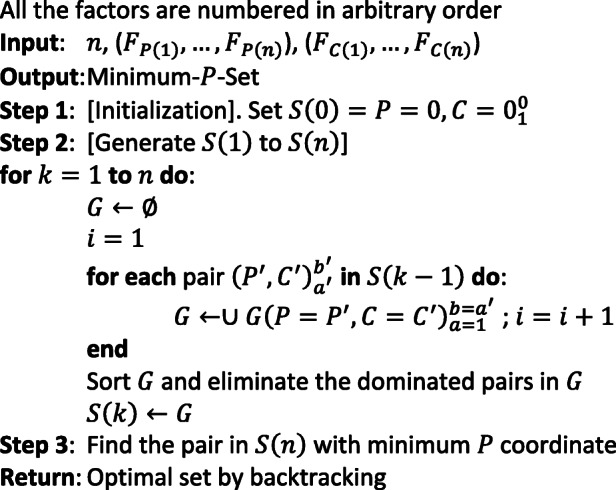


### Case study

We carried out a case study to put our factor selection method into practice. The data and observations used in our analysis were collected in 2012 from the ED at Bnai Zion hospital in Haifa, one of Israel’s largest cities. According to the Israel Ministry of Health [16, 17], the Bnai Zion ED admitted 62,900 patients in 2012, the patient’s mean length-of-stay in the ED was 2.84 h, and the median was 2.27 h. The number of patients admitted to the ED and patients’ length-of-stay are expected to have grown in correlation with the growth of the general population. The acute section of the ED contains 25 beds, and in the ambulatory section there is enough space for 25 people to sit. In every shift there are 4 physicians, 7 nurses (1 or 2 nurses in triage, and the others in other parts of the ED), and 2 receptionists. Each shift is 8 h long. Note that based on [17] and our observations there was no overcrowding situation at the Bnai-Zion ED during this study, a fact that aid us to locate the different factors.

**Fig. 2:**
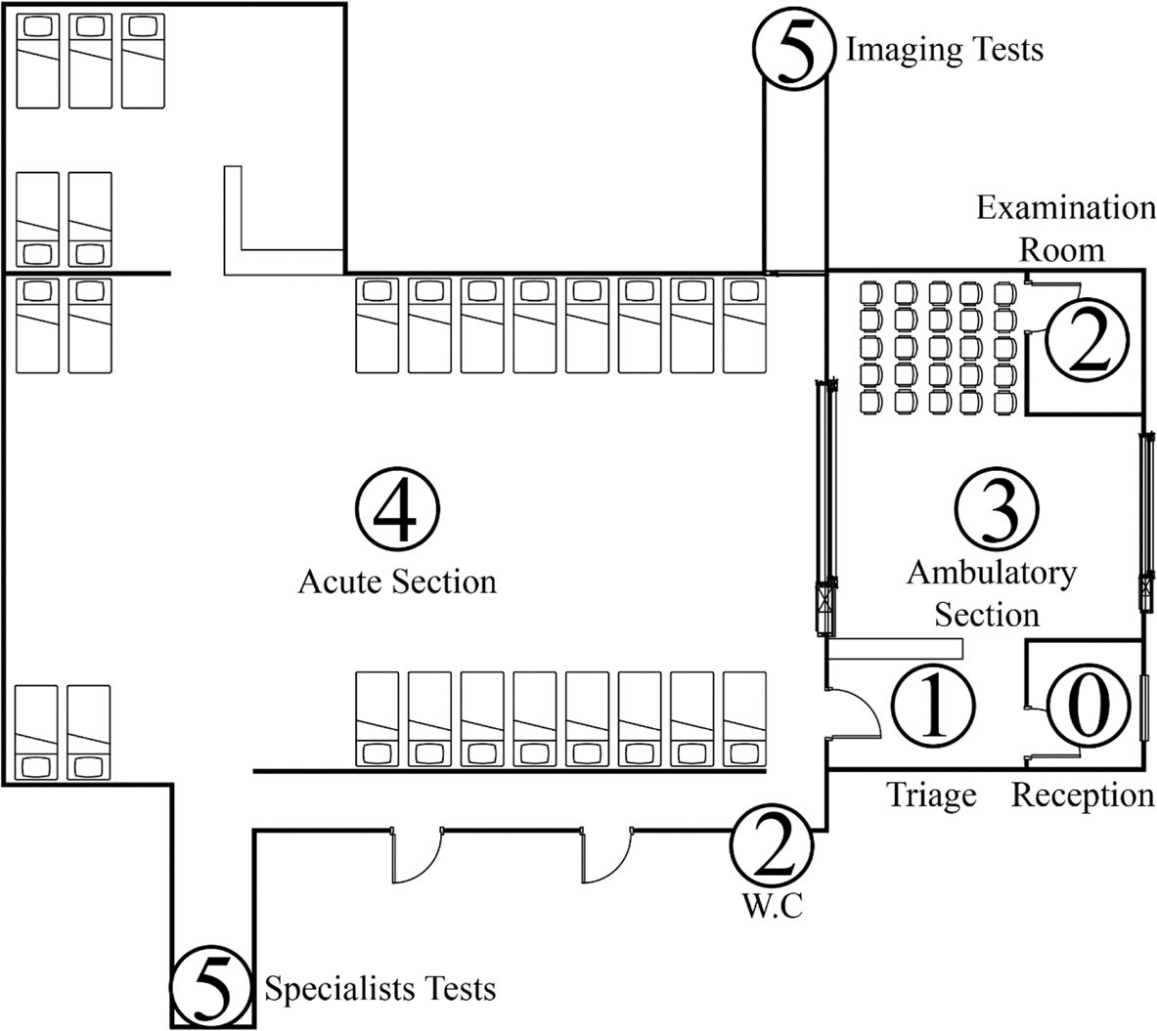
Floor Plan of ED. 0: Reception desk; 1: Triage; 2: Testing rooms; 3: Ambulatory section; 4: Acute section; 5: Additional testing

A patient who arrives at the ED is received by the receptionists (station 0 in Fig. 2). Triage (station 1) is the first station in the process chain. The triage nurses treat non-urgent patients and talk with each patient to obtain all available medical information; they check the patient’s body temperature, blood pressure and other general signs. Note that the nurse assigned at this point a severity index, similar to the ESI in purpose, in range of 1 to 10 where 1 is the lowest pain rate and 10 is the highest. The patient may then be sent to undergo testing such as blood tests and urinalysis (carried out at station 2). Once these results are obtained, a physician examines the patient (station 3: ambulatory section; station 4: acute section). The physician may request additional tests (station 5; performed outside the ED), provide treatment and wait to see the results, refer the patient for further treatment at home (with the general practitioner or a specialist), or refer him or her to one of the departments at the hospital.

### Data collection

We used data from the ED’s management software, patients’ record files, and observations. The data from the management software comprised information on 711 patients who arrived during various hours during a week in July 2012. For each patient, the following information was available: diagnosis and treatment codes, gender, age, date, arrival and release hours, case I. D, responsible department, arrival reason, an indicator reflecting whether the patient was hospitalized or released, hospitalization department (in the case of the former), and an indicator reflecting whether the patient left without receiving any treatment.

Finally, we carried out real-life observations on 100 different patients from the 711 random patients (gender, age, etc.) during various hours (day and night) throughout the research period. Specifically, we observed each patient from the moment he (or she) arrived, recording his movement and impressions regarding the demeanor of the patient and his escort from the time the patient completed the registration and until the time he was released from the ED, either to go home or to transfer to a different department in the hospital. The data we observed per individual included the following: date, arrival hour, release hour, number of escorts and demeanor. In addition, we recorded every movement of the escorts, patients and staff from different parts of the ED, triage, and outside of the ED. We also talked to the ED manager and some of the nurses, as it seemed reasonable that ED staff members might be able to point out phenomena that influence their work flow that might have escaped our notice otherwise. We also had access to the 100 real-life observed patients’ record files from the hospital archives. These were hand-written by the physicians and triage nurses, and included the following: body temperature, heart rate, blood pressure, severity index, blood tests, urinalysis and imaging tests (ECG, CT, X-ray); no identifying data (i.e. names and i.ds) was observed, as we had to preserve the patients’ anonymity. Those real-life observations and archived records mining are usually not feasible in researches due to the time and approvals that are needed to do so. All the observed data (real-life, archived and management software data) were combined and synchronized to analyze them. no demographic data was observed in order to preserve the patients’ anonymity.

In order to find new phenomena and factors to analyze, we closely examined our observations regarding ED movement and identified the following four types of movement:

- Medical staff: Physicians and nurses usually move between the patients and the triage and usually do not leave the ED.
- Non-medical staff: These staff members are divided into two groups: ED support staff, and general staff such as clerks and cleaners. The ED support staff are mainly patients’ transporters who assist patients who cannot walk, transporting them from the ED to other departments in the hospital or to undergo tests in departments located outside the ED.
- Patients: Patients move between stations according to the process chain described above.
- Escorts: Our observations suggest that escorts’ movement and purpose have an important and negative role in the ED workflow. This might seem surprising, as escorts have no active task in the process chain; they simply accompany and support patients as they move from station to station. Yet consider an analogy of cars on a road: if each car was escorted by one or more cars, the road would become blocked very quickly. In addition to contributing to crowding, an escort can have additional effects: For instance, he/she may become unquiet or impatient and, even unintentionally, disturb the medical and professional staff. This increases the workload and pressure in the ED. It is the reason why some hospitals in Israel have limited these last years each patient to one escort.

All the empirical data were sorted and arranged in Microsoft Excel 2013 and analyzed using IBM SPSS Statistics 20 software. We had full data (management system data and observations from the ED) for 100 patients, including handwritten files for those patients. We developed and compared statistical models to explore the impact of each of the observed variables on the dependent variables, i.e., patients’ length-of-stay (measured as the number of minutes between the patient’s arrival at the ED and his or her release from the ED) and crowding in the ED (measured as the number of people - including staff members, patients, and escorts - who are present in the ED at a given time). Specifically, we developed regression models using stepwise least squares and performed Pearson’s correlation tests on all the data, including only significant proven factors in order to avoid bias.

## Results

### Identification of significant factors

**Fig. 3:**
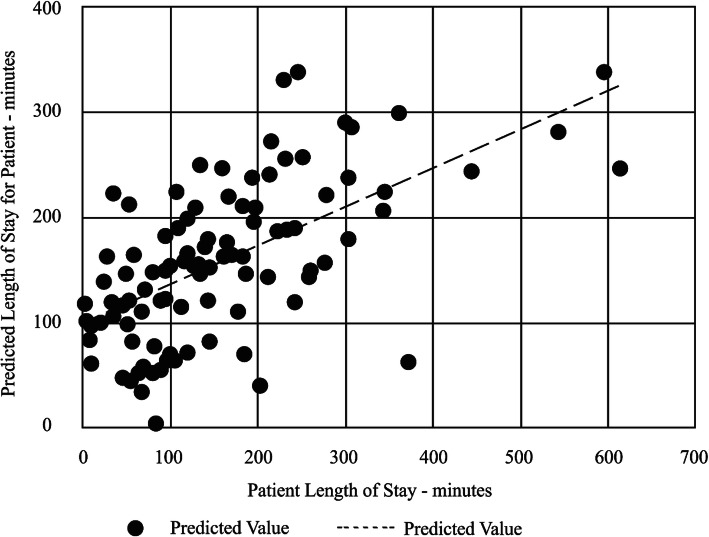
Relationship between actual LOS and predicted LOS based on regression model shown in Eq. 2

We examined the influence of all the factors mentioned above on patient length-of-stay and found that the following had significant effects: gender, age, hour of arrival, the number of people present in the ED overall and in specific areas of the ED (at the time of the patient’s arrival), the number of staff members present at the time of the patient’s arrival, the number of imaging tests (such as CT and X-ray) and regular tests (blood test and urinalysis) that the patient was asked to undergo, severity index, and the number of escorts who arrived with the patient. Using correlation tests in IBM SPSS, we found that the number of escorts accompanying a patient is the variable most strongly correlated with length-of-stay (*R*^2^ value of 0.362). In addition, we used SPSS Model Builder to develop a regression model to evaluate which variables predict patient length-of-stay. We identified the following six variables (Eq. 2, Table 1, Fig. 3), presented in order of significance: number of escorts arriving with the patient, heart rate in initial examination, number of escorts in the acute section at the time of the patient’s arrival, number of patients in the ambulatory section at the time of arrival, hour of arrival, and the number of non-imaging tests needed (blood tests, urinalysis).

2 $$ LOS=55.193\times {E}_p+3\times HR-6.303\times {E}_a+5.045\times {P}_a+5.716\times {H}_a+80.607\times {T}_{ni} $$

**Table 1:** Variables predicting length-of-stay

Source		Sum of Squares	df	Mean Square	F	Sig.	Coefficients
Corrected Model		523,408	6	87,235	8.665	.000	
Escorts per patient	*E*_*p*_	294,725	1	294,725	29.274	.000	55.193
Heart Rate (BPM)	*HR*	140,500	1	140,500	13.955	.000	3
Escorts in acute section	*E*_*a*_	83,800	1	83,800	8.324	.005	−6.303
Patients in ambulatory section	*P*_*a*_	62,734	1	62,734	6.231	.014	5.045
Hour of arrival	*H*_*a*_	48,698	1	48,698	4.837	.030	5.716
Tests needed	*T*_*ni*_	24,937	1	24,937	2.477	.119	80.607
Residual		896,030	89	10,067.750			
Corrected Total		1,419,438	95

As noted above, the number of escorts arriving with a patient had a significant influence on the patient’s length-of-stay; each additional escort was estimated to add 55 min, on average, to the patient’s length-of-stay. Surprisingly, the number of escorts present in the acute section had a significant negative effect on length-of-stay: Each additional escort present in the acute section upon a patient’s arrival was estimated to decrease the patient’s length-of-stay by 6 min. Each additional patient in the ambulatory section was associated with an increase of 5 min in an incoming patient’s length-of-stay. Another surprising observation is that the severity index was not found to be a significant factor regarding the LOS. It may be surprising but due to its characteristics it makes some sense, as we can assume that low severity index and high severity index will have shorter LOS in the ED. Finally, heart rate also had a significant impact on length-of-stay; each additional beat per minute (BPM) was estimated to be associated with 3 additional minutes.

We also carried out a separate analysis to evaluate how various factors influence crowding in the ED. For this analysis, we measured crowding, as well as the values of the factors of interest, concurrently at specific points in time: namely, whenever a movement (of any of the four types described above) was observed in the ED. The factors examined for this analysis were: hour in the day (“day or night” shifts), the number of staff members present in the ED, the number of tests requested per patient, and identity of the physician in charge. In addition, on the basis of patients’ files, we associated each patient’s time of arrival with the name of the department to which the patient was ultimately referred (“responsible department”).

We found that the variable “day/night” was the variable most strongly correlated with crowding (with a Pearson’s correlation value of 0.695). The number of tests assigned to patients (blood tests or urinalysis) was also correlated with crowding (Pearson’s correlation value of 0.635). In addition, as we did for length-of-stay, we developed a regression model using the SPSS Model Builder. We found only three variables that were predictive of crowding in the ED (with 52.3% accuracy, excluding variables that have an obvious influence, e.g., number of escorts; Eq. 3, Table 2). These variables included (in order of significance): “day/night”, responsible department, and number of tests (blood-test / urinalysis).

3 $$ Crowding=16.217\times D+7.22\times {D}_R+10.521\times {T}_{ni} $$

**Table 2:** Variables predicting crowding

Source		Sum of Squares	df	Mean Square	F	Sig.	Coefficients
Corrected Model		8165.534	4	2041.383	27.052	.000	
Day / Night	*D*	5475.415	1	5475.415	72.558	.000	16.217
Responsible department	*D*_*R*_	772.756	2	386.378	5.120	.008	7.22
Tests	*T*_*ni*_	210.405	1	210.405	2.788	.098	10.521
Residual		6867.091	91	75.461			
Corrected Total		15,032.625	95				

These results may seem intuitive; nevertheless, the “tests” variable had a significance of 0.098 (90.2%). This may indicate that an increased requirement for tests leads to crowding, or that crowding leads to a greater likelihood that patients will be requested to undergo tests.

### Implementation of the algorithm

For this stage of the case study we focus on factors influencing patients’ length-of-stay. As shown in Table 1, we found 6 factors that significantly affect this variable. Based on staff interviews, we assume that, in the best-case scenario, the average length-of-stay can be reduced by at most 2 h. The potential for reduction is obtain from the statistical analyses presented earlier, and the cost corresponding to each factor is subjective (Table 3), derived from information obtained from interviews with staff. In particular, the cost is an estimation integrating the financial costs and effort associated with implementing the respective factor. We assume that the maximal budget is 500 points (subjective).

**Table 3:** Algorithm’s input - factors influencing patients’ length-of-stay

Factor	Source		Potential reduction (minutes)	Steps required to address the factor	Responsible	Cost
1	Escorts per patient	*E*_*p*_	55	Adding a security guard	Management	100
2	Heart Rate (BPM)^a^	*HR*	30	Calm anxious patients	Physicians / nurses	20
3	Escorts in acute section	*E*_*a*_	6	Only 1 escort per patient	Nurses	30
4	Patients in ambulatory section	*P*_*a*_	5	Increase treatment speed	Management	500
5	Hour of arrival	*H*_*a*_	6	Impossible to control	Management	Infinity
6	Tests needed	*T*_*ni*_	80	Avoid unnecessary tests	Nurses	30

^a^Note: We assume that the average patient’s heart rate can be reduced by 10 BPM

Addressing the number of escorts per patient can be achieved by putting a signboard and adding a security guard to the entrance of the ED, or even reassigning a security guard who is working in a different location. Heart rate reduction can be achieved, for example, by providing ED medical staff with guidance on how to identify patients who are anxious and help them calm down. (We assume that the average patient’s heart rate can be reduced by 10 BPM.) The escorts in the acute section have a negative effect on patients’ length-of-stay (Table 1), which means that patients in the acute section benefit from the presence of escorts. Nevertheless, there should be a balance between *E*_*p*_ and *E*_*a*_; therefore, a policy of one escort per patient can be implemented under the supervision of the nurses. The factor of “patients in ambulatory section” is difficult to address and is likely to require a large quantity of resources. We therefore assume that the cost of addressing this factor is 500, i.e., the full extent of our budget, which means that if the management decides to address this factor, no other factors can be addressed at the same time. Last, the “tests needed” factor can be addressed by guiding triage nurses to avoid sending patients to unnecessary tests.

To decide which factors ED managers should address, we ran the algorithm with the settings outlined in Table 3. At each stage we checked the addition of the next factor to the set of factors already checked. First, we initialized the set of subsets *S*(0) with no cost or potential for LOS reduction. Then, we added the first factor as shown in Table 4, which represents the set of subsets *S*(1).

**Table 4:** *S*(1)

Factors	P	C
0	0	0
0–1	55	100

For an example of how the algorithm works, Table 5 (corresponding to *S*(3)) shows the elimination rule: Subset 0–3 is dominated by subset 0–2, as the former costs more and has less potential for LOS reduction.

**Table 5:** *S*(3)

Factors	P	C
0	0	0
0–3	6	30
0–2	30	20
0–2-3	36	50
0–1	55	100
0–1-3	61	130
0–1-2	85	120
0–1–2-3	91	150

The algorithm indicates that the optimal set of factors to treat in our case comprises factors 1, 2, 3, and 6, namely, the number of escorts per patient, patients’ heart rate, number of escorts in the acute section, and the number of tests needed.

## Discussion and managerial implications

Our analysis points to several factors that may influence patient length-of-stay and crowding in the ED. One such factor is the number of tests (blood test and urinalysis) required to be performed per patient. This observation might reflect situations in which triage nurses, faced with a large group of incoming patients, have less time to deliberate about diagnoses and therefore send more patients for tests. Alternatively, testing might be used a means to “buy more time” for the ED staff to handle patients; this method might reduce pressure on staff but increase crowding and individual patients’ length-of-stay. We carried out interviews with physicians and nurses in order to gain a better understanding of the meaning of this finding, and the prevailing opinion was that this phenomenon might be specific to the observed ED. Nevertheless, it is recommended to establish a clear procedure in order to prevent disorder and to control this factor.

We further observed that the number of escorts accompanying a patient has a substantial influence on length-of-stay. Notably, the role of escorts in ED patients’ length-of-stay has yet to be discussed in the literature. We found that every additional escort accompanying a patient was associated with an increase of 55 min in the patient’s length-of-stay. That influence might be associated with the additional demands or distractions that escorts pose on staff members or on patients. On the basis of our findings, we recommend that ED management should restrict the number of escorts to one per patient. This method has been practiced in various hospitals but has not been empirically examined until now. The case-study’s ED is located at the entrance to the hospital, which means that the management can use hospital security staff to enforce the limitation on escorts without requiring a substantial increase in resources. Another available method for addressing this factor is the use of BLE or RFID tags, similar to those that patients get in some EDs to help monitoring the escorts and their numbers; another low-tech method that is currently in use in Israel is the use of bracelets or stamps for each patient and his escort. Following our above recommendation, the management of the current ED has agreed to adopt and applicate the suggestion regarding escort limitation and reported quite satisfactory results. In the recent years, more EDs in Israel have adopted the one escort per patient recommendation too and use it currently.

Another new factor we identified is the patient’s heart rate upon arrival. An elevated heart rate may be caused by stress due to the presence of medical staff (the well-known “white coat hypertension”, see [24, 31, 12]) or may be directly related to the patient’s medical condition. In any case, taking the time to assess that the hypertension is not caused by the white coat effect may give good results and will not worsen the situation. The association between heart rate and length-of-stay may reflect a need to keep patients with an elevated heart rate under supervision, or a need to devote more time to speaking with, diagnosing and treating such patients.

More generally, and given that the time of day was shown to have a significant effect on crowding in the ED, it is possible that the triage station might benefit from reformulation of procedures, particularly with regard to periods of high pressure in the ED. It is possible that it would be sufficient just to encourage nurses to prepare in advance for high-pressure times of day, and that this would reduce the number of unnecessary tests. Alternatively, ED managers could reconsider the scheduling approaches used at high-pressure times. For example, at high-pressure times, it might actually be advantageous to ask all patients to undergo blood tests and urinalysis, as in a conveyor belt. This approach might decrease examination time at the triage enough to compensate for the lack of efficiency involved in testing all patients. This could be a fruitful topic for future scheduling-and-control research that incorporates elements from psychology.

The reader needs to keep in mind that those results were obtained in a single ED. There may be a non-negligible variability between EDs, between hospitals in Israel and between different healthcare systems all around the world. Although those results were significant, there is a strong possibility that they will be found insignificant in other EDs with other settings, or that the factors we dealt with will not be the most efficient to treat there. We firmly believe that it is of highest importance to get good and relevant data from each specific ED and system and to analyze it properly in order to achieve such results that will improve the ED performances, as we did here.

## Conclusions

Our goal was to show that by collecting data inside the ED - including analysis of data from information systems, patient files, real-time observations and conversations with ED staff - we can identify new factors that have a significant influence on patient length-of-stay or on crowding in the ED. We subsequently proposed and applied an algorithm to assist ED managers in selecting the factors that they would benefit most from addressing, given budgetary constraints. We note that this algorithmic method can be applied even in the absence of data, e.g., when factors are selected solely on the basis of physicians’ experience. Application of the proposed statistical and algorithmic method is straightforward and should be feasible for most small and medium hospitals. It can be used for periodic planning of resources. Our approach takes into account the workload and financial constraints involved in implementing change, such that it can be applied even when resources are limited. The study was made during the summer of 2012, regardless we found factors that were not taken into account at that time and are currently considered to have large influence on the workload at the EDs. Future research may investigate further our findings and also address many factors that were not yet available in the ED’s information systems during the original research. A thorough multivariate analysis of all the studied factors as well as newly available ones may give good results and insights.

It is interesting to note that our proposal for limitation of escorts number to one per patient has been adopted in Bnai Zion and became during the years a common rule or recommendation in most of the EDs in Israel; a check done in 2019 found that 12 of the 18 biggest EDs in Israel applied it, for the rest no strict instructions were published. EDs may look for effective solutions to monitor the number of visitors and their locations (i.e., if they should stay only in the waiting area and or help with tests in the care area) also by the application of technological innovations. BLE tags and Wi-Fi sensors in the ED can validate that only approved visitors enter specific areas. Another simple solution is the use of RFID tags that are cheep and easy to apply in a bracelet to the patient and the escort. Addressing some of the factors using new technology and innovations may improve the results and the ways to apply them and should be studied further in order to improve the patients’ LOS in the EDs.

An additional factor was the contribution of the white coat effect to the heart rate of the patient that has been admitted in the ED, and hence to the LOS. This effect can be reduced at least partially by encouraging the patient to ask questions and the physicians to answer them and reduce the tension; this may be done through a proper training of the medical staff regarding this subject. Future research may study more thoroughly the influence of this factor in the EDs.

Those facts emphasize the importance of our approach to be applied in the EDs and additional departments in general. We wish also to recommend the introduction and checking of our planning method in further EDs where the budgets limitations are very strict. We have further proposed several specific, practical means of overcoming the bottlenecks we identified at the Bnai Zion ED. Appropriate resolution of these bottlenecks has the potential to reduce patient length-of-stay and crowding in the ED. It would be of interest for future studies, especially empirical ones that will be applied to other hospitals, to identify the actual effects of addressing such bottlenecks, as well as to use real-life analysis to gain further insight into the mechanisms by which various factors affect ED crowding and patient length-of-stay.

## Data Availability

‘Not applicable’.
